# Antitubercular drug poisoning in a pregnant woman

**DOI:** 10.4103/0019-5049.68383

**Published:** 2010

**Authors:** Rahul Dutta, Surya Kumar Dube, Dinesh Kumar Singh

**Affiliations:** Department of Anaesthesiology, Institute of Medical Sciences, Banaras Hindu University, Varanasi - 221 005, India

**Keywords:** Acute-intoxication, antitubercular, pregnancy, pyridoxine

## Abstract

A 20-year-old female in her third month of pregnancy, presented with generalised tonic clonic seizures, metabolic acidosis and coma following suicidal ingestion of antitubercular medication. We successfully managed the case with pyridoxine, sodium bicarbonate and mechanical ventilation.

## INTRODUCTION

Isoniazid (INH) is a major component of both therapeutic and prophylactic antitubercular therapy. Over dose of Isoniazid usually manifests with mild neurological side effects like peripheral neuritis, dizziness, and insomnia.[1] However, acute ingestion of INH at a high dose can cause recurrent seizures resistant to anticonvulsant therapy, profound metabolic acidosis, coma and even death.[2,3] Pyridoxine administered in a dose equivalent to the suspected maximum amount of isoniazid ingested can potentially revert the entire clinical and metabolic outcome resulting from isoniazid intoxication. Here we report a successful management of a 20 year pregnant women presented to us with isoniazid poisoning.

## CASE REPORT

A 20-year-old female in her third month of pregnancy presented to us following 3-4 episodes of vomiting and unconsciousness. On presentation, her Glasgow Coma Scale was 10/15 and was found to have heart rate and blood pressure of 110/min and 110/55 mmHg, respectively. Her respiration was spontaneous and regular at the rate of 18 breaths/min. Her pupils were normal in size and reacting to light and neck rigidity and Kernig’s sign were absent. Bilateral chest was clear on auscultation. Following admission, she had generalised tonic clonic convulsions which was unresponsive to 4 mg Intravenous (IV) Lorazepam. Immediately Inj. Thiopentone was given and her airway was secured with a tracheal tube. Her initial arterial blood gas (ABG) was as follows: pH - 6.86, PaCO_2_ - 72 mm Hg, PaO_2_ - 108 mm Hg, HCO_3_ ^-^ 12.7, Base deficit - 21.3, Na^+^ - 132 meq/l, K^+^ - 3 meq/l, Ca^2+^ - 9.3 mg/dl and glucose of 200 mg/dl. Immediately, she was provided with 100 meq of sodium bicarbonate (7.5%) and was put on controlled mandatory mode ventilation with FiO_2_ 0.5, tidal volume 400 ml and respiratory rate of 20 breaths per minute. The ventilatory settings were periodically adjusted to achieve normocapnoea and normooxaemia. We corrected the hypokalemia with intravenous inj. potassium chloride at the rate of 20 meq/hr. Renal function test, coagulation profile and urine examination of the patient were within normal limits. Liver function test of the patient showed an ALT (Alanine transferase) and AST (Aspartate transferase) of 63 IU/L and 83 IU/L, respectively, with out any other abnormalities.

Detailed history revealed that the patient was a diagnosed case of pulmonary tuberculosis and was on antitubercular therapy and had consumed nearly 10 tablets of some medication. We strongly suspected an acute intoxication of isoniazide (due to the clinical presentation) and immediately gastric lavage was done with 100 g charcoal mixed with 50 g of sorbitol through a nasogastric tube. Intravenous pyridoxine (3,000 mg) was then administered slowly over 15 minutes. After three hours, her ABG was: pH - 7.41, PaCO_2_ -31 mmHg, PaO_2_ - 109 mmHg, HCO_3_-18.7, Base deficit - 4.9. Twelve hours afterward her blood gases were: pH - 7.43, PaCO_2_ - 36 mmHg, PaO_2_ - 105 mmHg, HCO_3_^−^ - 23.6, Base deficit - 1.0. On second day, she regained consciousness and her blood biochemistry, cerebrospinal fluid examination, coagulation profile and blood gas parameters were within normal limit. Ultrasound abdomen for assessment of fetal status was within normal limit. Patient was extubated on the second day. On day three, her liver function test (ALT and AST) were within normal limit and patient was discharged from the ICU.

## DISCUSSION

Presentation of metabolic acidosis with hypokalemia is seen in conditions like diabetic ketoacidosis, hepatic failure, poisoning like isoniazide, ammonium chloride and toluene, and some hormonal disorders like that of mineralocorticoid and glucocoticiod. The possibility of diabetic ketoacidosis and hepatic failure had been ruled out from the blood sugar level, normal urine analysis and liver function tests. There was no family or personal history of any endocrinal disorder. So we ruled out the possibility of any endocrinopathy. As there was a history of intake of some medications and the patient was a case of pulmonary tuberculosis, we focused our diagnosis and subsequent management on antitubercular drug poisoning. Normally physiological changes occur in respiratory system during pregnancy under the influence of increasing progesterone level. There is a progressive increase in the minute ventilation (50%) and oxygen consumption during the antenatal period. The increase in minute ventilation is more because of increase in tidal volume (40%) and small contribution is from increase in respiratory rate (15%). But these changes are seen in case of normal pregnancy. Our case is of metabolic acidosis with a respiratory rate of18 breaths/min and had recent seizures. Severe metabolic acidosis can cause cardio-respiratory depression. This can explain the higher PaCO_2_ in this patient.

Isoniazid (INH) is a major component of both therapeutic and prophylactic antitubercular therapy. Isoniazide over dose usually manifests with mild neurological side effects, including peripheral neuritis, dizziness, and insomnia.[1] However, acute ingestion of INH in doses of 30 mg/kg or more can produce seizures which are usually resistant to anticonvulsant therapy. On ingestion in amount greater than 80 mg/kg, recurrent seizures, profound metabolic acidosis, coma and even death can occur.[2,3]

The first sign and symptom of isoniazid toxicity may appear within thirty minutes to two hours after ingestion and may include nausea, vomiting, rash, fever, ataxia, slurring of speech, peripheral neuritis, dizziness and stupor. These symptoms are usually followed by grand mal seizures and coma. The seizures are often refractory to anticonvulsants, particularly phenytoin and barbiturates. INH is thought to cause seizures by interfering with γ-aminobutyric acid synthesis.[4] Specifically, INH inhibits glutamic acid decarboxylase[5] by inhibiting pyridoxal 5- phosphate which is a co-factor for glutamic acid decarboxylase enzyme. The consequent reduction in GABA (γ-aminobutyric acid) level increases the susceptibility to seizure. Pyridoxine administration can potentially revert the entire clinical and metabolic outcome resulting from isoniazid intoxication.[6,7] Pyridoxine should be administered in a dose equivalent to the suspected maximum amount of isoniazid ingested (i.e., gram-per-gram replacement).[8] If the amount of ingested isoniazid is unknown, 5 g of pyridoxine is given intravenously over 5 to 10 minutes. Repeat dosing may be needed for persistent seizure activity and may also be used to reverse deep coma.

In adults, gastric lavage should be carried out with slurry of charcoal (1-2 g/kg) mixed with sorbitol (1 g/kg) and if needed to be repeated with charcoal only.[9] As, activated charcoal is not systemically absorbed from the gastrointestinal tract it’s use is considered safe in pregnant patient.[10] Acidosis should only be corrected with sodium bicarbonate in dose of 1-3 meq/kg. if the pH values are less than 7.1.[11] Haemodialysis and peritoneal dialysis removes isoniazid from serum and should be carried out if pyridoxine and thiopentone are not able to control the seizures or the serum levels are quite high.[12] Rhabdomylosis is an uncommon but potentially lethal complication of acute isoniazid intoxication.[13]

Rifampicin intake is not associated with serious adverse reactions. Minor side effects like nausea and vomiting can be managed with symptomatic treatment. Rarely, major adverse reactions endangering life such as hemolysis, shock, thrombocytopenia and acute renal failure may develop.[13]

## CONCLUSION

With the increasing incidence of tuberculosis and use of antitubercular drugs, a diagnosis of acute intoxication of antituberculosis drugs should always be considered if a patient presents presenting with clinical triad of seizures not responding to anticonvulsant, metabolic acidosis and coma. Aggressive treatment of seizures with intravenous pyridoxine could potentially counteract the fatal toxic effects of isoniazid.
